# High-Commitment Human Resource Management and Care Workers’ Attitudes and Performance Through a Sustainable-Careers Lens: A Multilevel Analysis in Japan

**DOI:** 10.3390/bs16081369

**Published:** 2026-08-10

**Authors:** Masako Sugano

**Affiliations:** Department of Business Administration, College of Business Administration, Ibaraki Christian University, 6-11-1 Omika-cho, Hitachi, Ibaraki 319-1295, Japan; m.sugano@icc.ac.jp; Tel.: +81-294-52-3215

**Keywords:** high-commitment human resource management, ability–motivation–opportunity theory, work–life balance support, sustainable careers, coworker exchange, hierarchical linear modeling, long-term care, Japan

## Abstract

In aging societies, securing a stable long-term care workforce is a priority, yet workforce policies centered on financial incentives have demonstrated limited effectiveness. Drawing on the sustainable-careers perspective as an analytical lens, this study re-examines high-commitment human resource management (HCHRM), grounded in ability–motivation–opportunity (AMO) theory, by investigating how it relates to the work attitudes and work role performance of long-term care workers, with work–life balance (WLB) support incorporated as an opportunity-enhancing practice. Data of 875 full-time care workers from 130 establishments in Japan were analyzed using hierarchical linear modeling. Opportunity-enhancing practices (WLB support and autonomous team management) were positively associated with affective commitment, and coworker exchange (CWX) was favorably associated with all outcomes. Cross-level analyses indicated that higher WLB support was associated with a stronger relationship between CWX and both lower turnover intention and greater proficiency behavior, whereas higher autonomous team management was associated with a stronger CWX–adaptive-proactive behavior relationship. Interpreted through a sustainable-careers lens, these findings position WLB support as an opportunity-enhancing practice within AMO theory and indicate that it may operate synergistically with workplace relationships to support care workers’ attitudes and performance.

## 1. Introduction

Across sectors, technological advancement, including artificial intelligence and automation, is reshaping how organizations manage their workforce. However, in long-term care, the relational nature of care work means that technology is more likely to complement care workers than to replace them (OECD, 2020). Therefore, ensuring care quality and retaining a stable workforce depend heavily on human resource management (HRM), including training, improved working conditions, and support for motivation (OECD, 2020).

The shortage of long-term care workers driven by the rapid population aging is a challenge across advanced economies. In response, many countries have implemented policies centered primarily on financial incentives, such as wage increases (OECD, 2020). Japan is no exception, with the national government continuously implementing treatment improvement measures (Ministry of Health, Labour and Welfare, 2015, 2017). Although such measures have merit, they are insufficient for reducing turnover and sustaining motivation, and multifaceted approaches (e.g., improvements in the work environment and investment in training and development) are necessary (OECD, 2020). A review of Japan’s long-term care sector by Takeda (2023) identified workplace interpersonal relationships as a key predictor of turnover intention.

Previous research in healthcare and social welfare has examined high-commitment human resource management (HCHRM), grounded in ability–motivation–opportunity (AMO) theory, as a framework for improving outcomes through employee commitment, with evidence supporting its effectiveness (e.g., Renkema et al., 2021; Schopman et al., 2015). In long-term care, Hasenfeld (1983, 2010), who established the foundational framework of human services organizations, argued that care quantity and quality depend not only on knowledge, skills, and motivation but also on empowerment through on-the-ground discretion and mutual adjustment, making AMO theory well suited to this sector.

However, several research gaps remain. First, much of the HCHRM literature relies on single-level analysis, leaving underexplored how organizational practices interact with individual experiences and workplace relationships (Harley et al., 2007; Renkema et al., 2021; Rondeau & Wagar, 2001; Schopman et al., 2015). Although HRM practices are implemented at the organizational level, their effects materialize through employees’ perceptions and behaviors (Wright & Nishii, 2006). Data reflecting this organizational–individual hierarchy and analyses that separate these levels are therefore essential. Second, many studies treat AMO components as a bundled construct, without adequately examining the effects of individual practices. Particularly, opportunity-enhancing practices (i.e., environmental conditions that enable ability and motivation to be realized) remain underexamined in terms of content and mechanisms. Third, most HCHRM studies emphasize performance improvement, with limited attention to “sustainable careers”—a perspective that foregrounds the long-term continuity of the employment relationship and workers’ capacity to sustain and adapt their careers over time (De Vos et al., 2020; Van der Heijden & De Vos, 2015). Fourth, despite the consistent emphasis on leadership and teamwork in long-term care (de Bruin et al., 2022; Harley et al., 2007; Renkema et al., 2021; Rondeau & Wagar, 2001; Schopman et al., 2015), research integrating workplace relationship quality, including leader–member exchange (LMX) and coworker exchange (CWX), into HCHRM models remains limited. Notably, studies incorporating CWX are largely absent, and the moderating role of opportunity-enhancing practices in these relationships remains unexplored.

Accordingly, this study aims to examine how AMO theory-grounded HCHRM relates to work attitudes and work role performance among long-term care workers, through a sustainable-careers lens. Multilevel analysis is employed to statistically disentangle organizational- and individual-level variables and examine cross-level effects between the two. Among AMO-based HCHRM practices, work–life balance (WLB) support is incorporated as an opportunity-enhancing factor alongside the autonomy and discretion examined in previous research, as a means of supporting care workers’ sustainable careers. This study examines both the direct associations of WLB support and its cross-level moderating role in relation to social resources (LMX and CWX). In this framework, LMX and CWX represent individual-level social resources, while opportunity-enhancing practices function as organizational conditions that enable these resources to translate into outcomes.

This study makes two important contributions. First, it examines HCHRM through a sustainable-careers lens and its associations with care workers’ work attitudes and work role performance using hierarchically structured data across organizational and individual levels. Accordingly, it extends HCHRM research beyond performance toward long-term employment sustainability and adaptability. Second, by positioning WLB support as an opportunity-enhancing practice and empirically examining its direct associations and moderating role in relation to LMX and CWX, it provides practical implications for the design of HR practices aimed at workforce retention and development in long-term care settings.

## 2. Literature Review

### 2.1. HCHRM and AMO Theory

HCHRM is a human resource management model that enhances employees’ affective commitment to the organization through ability development and motivation, thereby promoting discretionary behavior (Guest, 1997). It developed within the field of strategic human resource management, which examines the strategic role of HRM in organizational outcomes (Wright & McMahan, 1992). A related concept is “high-performance work systems” (Huselid, 1995); however, this study adopted the HCHRM framework, which centers on enhancing organizational commitment. This focus reflects the nature of long-term care work as a human services profession, where performance depends heavily on affective dimensions such as value internalization and service dedication (Eaton, 2000; Hasenfeld, 1983, 2010). These foundational works collectively shaped the understanding of how HRM practices enhance employee commitment and organizational performance—the core premise of the HCHRM framework adopted in this study—and remain central to contemporary strategic human resource management research (e.g., Kramar, 2022; Saridakis et al., 2017). Additionally, meta-analyses by Jiang et al. (2012) and Saridakis et al. (2017) demonstrate that HCHRM improves organizational commitment, reduces stress and turnover, and increases productivity. In long-term care, early studies demonstrated that HCHRM positively influences work attitudes and behavior (Harley et al., 2007; Rondeau & Wagar, 2001). These findings have been consistently supported by more recent research (Renkema et al., 2021; Schopman et al., 2015).

This study draws on AMO theory (Appelbaum et al., 2000; Blumberg & Pringle, 1982), the theoretical foundation of HCHRM that continues to inform contemporary HRM research (e.g., Bos-Nehles et al., 2023; Jiang et al., 2012). AMO theory posits that when HRM practices enhance employees’ ability, motivation, and opportunity, employee motivation and performance improve, strengthening sustained competitive advantage. As noted, care quantity and quality depend not only on knowledge, skills, and motivation but also on empowerment through discretion and mutual adjustment (Hasenfeld, 1983, 2010). Given the labor-intensive nature of long-term care, AMO-based frameworks are widely recognized as essential for organizational effectiveness because they develop ability (A), elicit motivation (M), and enable autonomous judgment and action (O) (Rondeau & Wagar, 2001; Schopman et al., 2015). Practice examples of AMO-based frameworks include (1) ability-enhancing practices, such as recruitment, selection, training, and development; (2) motivation-enhancing practices, including compensation, incentives, and performance management; and (3) opportunity-enhancing practices, like autonomy, discretion, and team development (Lepak et al., 2006). These elements are complementary and, when implemented as bundles, generate synergistic effects (Appelbaum et al., 2000; Boon et al., 2019; Lepak et al., 2006). Concomitantly, research has increasingly examined individual AMO components. A meta-analysis by Jiang et al. (2012) found that ability-enhancing practices strongly relate to human capital development, whereas motivation- and opportunity-enhancing practices more strongly relate to employee motivation. Furthermore, “opportunity” functions as an environmental condition enabling ability and motivation (Blumberg & Pringle, 1982), moderating their relationships with other outcomes (Bos-Nehles et al., 2023).

The usefulness of this AMO-based framework is particularly salient in long-term care. First, such services exemplify human services organizations (Hasenfeld, 1983, 2010), where care quality depends, given the diversity of recipient needs, on discretionary judgment rather than strict adherence to standardized procedures. Therefore, high ability and motivation do not translate into outcomes without an enabling opportunity environment. Second, teamwork is consistently identified as essential for care quality and employee outcomes (Harley et al., 2007; Renkema et al., 2021; Rondeau & Wagar, 2001; Schopman et al., 2015). Individual ability and motivation translate into organizational outcomes only when an opportunity environment, characterized by information sharing, role coordination, and mutual adjustment, functions effectively. Together, these points suggest that the opportunity component is an indispensable condition through which ability and motivation translate into outcomes, although, compared with ability- and motivation-enhancing practices, it has received relatively less empirical attention in long-term care. Accordingly, this study explores the opportunity component of AMO theory, examining its moderating effects within the contextual considerations outlined above.

### 2.2. Sustainable Careers and WLB Support

HCHRM has also been associated with a “dark side,” including unintended negative outcomes such as excessive working hours and deteriorating health (Van De Voorde et al., 2012; White et al., 2003). The long-term care sector faces not only structural resource constraints but also chronic staff shortages and elevated risks of stress and burnout (Hasenfeld, 1983, 2010; OECD, 2020). In this context, overly performance-driven HRM may deplete employees’ personal resources. This setting renders the concept of “sustainable careers” particularly relevant (De Vos & Van der Heijden, 2017; De Vos et al., 2020; Van der Heijden & De Vos, 2015).

Sustainable careers are defined by Van der Heijden and De Vos (2015) and reformulated in De Vos et al. (2020, p. 1) as “sequences of career experiences reflected through a variety of patterns of continuity over time, thereby crossing several social spaces, characterized by individual agency, herewith providing meaning to the individual.” Three features of this definition are central to this study. First, individual agency implies that career decisions rest with the individual, who actively shapes their own career. Second, crossing several social spaces situates careers across multiple life domains—including work, family, and personal life. Third, continuity over time emphasizes the sustained maintenance of an employment relationship rather than the maximization of short-term performance. This study reframes the conventionally adopted research design in performance-oriented HCHRM research through a sustainable-careers lens, focusing on the agency, work–life integration, and continuity on which sustainable careers depend. In long-term care, where stress and resource depletion are pervasive, sustaining a career requires not only preventing resource loss but also proactively acquiring and investing resources—a process that depends on employees being able to exercise autonomy over how they manage their work and personal lives. This highlights the importance of organizational conditions that enable such autonomy, the concept of which is developed below in positioning WLB support as an opportunity-enhancing practice.

Importantly, sustainable careers depend on both individual effort and HRM practices that actively support them (De Vos et al., 2020). De Prins et al. (2014) conceptualize sustainable HRM through the respect, openness, and continuity model. Continuity refers to sustaining employment relationships where employees maintain their health, motivation, and capabilities over time (regardless of age or circumstances) rather than maximizing short-term performance, aligning directly with sustainable careers. Kramar (2022) positions sustainable HRM as an extension or next stage of strategic human resource management, highlighting the integration of strategic and sustainability-oriented elements. Accordingly, examining HCHRM requires incorporating a continuity perspective, which should focus on long-term employment, health, and well-being, alongside the traditional performance emphasis. A broader shift has also been advocated from performance-oriented HRM toward well-being-focused HRM that generates mutual benefits for both employees and organizations (Guest, 2017). Therefore, for HCHRM to be supportive of sustainable careers, it should incorporate practices that actively align individual values with private life.

Against this backdrop, this study examines WLB support as an opportunity-enhancing practice within HCHRM. Haar et al. (2014) defined WLB as “an individual’s perceptions of how well their life roles are balanced,” showing that it improves work and life satisfaction while reducing mental health problems. Organizational WLB support refers to strategic HR practices that promote employees’ WLB (Haar et al., 2014), including flexible work arrangements and support for managing work and family responsibilities (Batt & Valcour, 2003). While WLB support has frequently been treated as secondary, scholars have called for its strategic integration into HCHRM in recent decades (Batt & Valcour, 2003; Perry-Smith & Blum, 2000).

The positioning of WLB support as an opportunity-enhancing practice in long-term care rests on the AMO premise that opportunity-enhancing practices are the environmental conditions that enable employees’ abilities and motivation to be exercised and translated into outcomes (Appelbaum et al., 2000; Bos-Nehles et al., 2023). WLB support functions as such a condition in three ways. First, from an AMO perspective, WLB support enables employees to manage work–life boundaries autonomously (Batt & Valcour, 2003; Thomas & Ganster, 1995), providing the discretion and control that allow their abilities and motivation to be exercised rather than being constrained by work–life conflict. Second, from a conservation of resources perspective, it facilitates resource recovery through rest and refreshment (Hobfoll et al., 2018), thereby restoring the physical and psychological resources that employees need to deploy their skills and sustain motivated engagement under the characteristic high-stress demands of long-term care settings. Third, from a continuity perspective, by helping employees remain in employment over time, it preserves the conditions under which their accumulated abilities and motivation can continue to be applied, rather than being lost through turnover or burnout. Across all three rationales, WLB support operates not by directly raising ability or motivation, but by shaping the conditions under which existing ability and motivation can be realized—the defining feature of an opportunity-enhancing practice. Accordingly, this study includes WLB support as an opportunity-enhancing practice alongside autonomy, discretion, and team development.

### 2.3. Work Attitudes and Work Behavior

This study examines work attitudes and work role performance as proximal, work-related outcomes that, viewed through a sustainable-careers lens, correspond to two concerns emphasized in that literature: (1) sustained employment over the medium to long term and (2) proactive adaptation to change. Work attitudes, specifically affective commitment and turnover intention, were treated as dependent variables representing the first outcome, while work role performance, referring here to proficiency, adaptive, and proactive behavior, served as dependent variables representing the second outcome. Affective commitment refers to an employee’s emotional attachment to, identification with, and involvement in the organization, promoting engagement driven by intrinsic motivation (Allen & Meyer, 1996). It serves as an indicator of “respect for persons” in sustainable HRM (De Prins et al., 2014). Meanwhile, turnover intention reflects an individual’s intent to leave their position (Mobley et al., 1979) and, viewed through a sustainable-careers lens, is relevant to career sustainability (De Prins et al., 2014; De Vos et al., 2020).

This study adopts the work role performance framework proposed by Griffin et al. (2007) to capture three forms of contributing behavior: proficiency behavior, referring to reliably fulfilling prescribed role requirements; adaptive behavior, referring to responding flexibly to changes in job systems and role demands; and proactive behavior, referring to anticipating change and taking initiative (Griffin et al., 2007). These behaviors, perceived through a sustainable-careers lens, relate to sustained productivity and career sustainability (De Vos et al., 2020).

### 2.4. LMX and CWX as Social Resources

In Japan’s long-term care sector, systematic HRM research remains limited, and findings on practice effectiveness are mixed (Haraguchi, 2015; Sugano, 2024; N. Takeuchi et al., 2007). Concurrently, the importance of interpersonal rewards for care workers has been widely recognized (Fujisawa & Haraguchi, 2016; So et al., 2007; Tao, 2001). Given the importance of interpersonal relationships in long-term care settings, this study examines workplace relationship quality as a social resource, exploring both LMX (Graen et al., 1982) and CWX (Sherony & Green, 2002). LMX refers to the quality of the supervisor–subordinate relationship, characterized by mutual affect, trust, and loyalty (Graen et al., 1982), and is consistently associated with positive work attitudes and behavior (Erdogan & Bauer, 2014). CWX refers to the quality of exchange relationships among coworkers who share the same supervisor and has also been shown to positively influence work attitudes and behavior (Liu et al., 2021; Sherony & Green, 2002). In team-based settings such as long-term care, CWX is particularly important because it captures mutual support and collaboration. Evidence from Japan indicates that coworker interaction is strongly associated with motivation and skill development, whereas LMX effects may be more limited (Sugano, 2020).

In this study, LMX and CWX were conceptualized as individual-level social resources and analyzed for their relationship with HCHRM, which warrants further clarification. Notably, HCHRM does not directly generate LMX or CWX, as these emerge through interpersonal interactions and may exist independently of HR practices. Accordingly, this study explains their linkage through the opportunity component of AMO theory. As discussed in Section 2.1, opportunity-enhancing practices function as organizational conditions that convert ability and motivation into outcomes (Blumberg & Pringle, 1982; Bos-Nehles et al., 2023). This study extends this logic to LMX and CWX, proposing that their effects on work attitudes and behavior depend on the organizational context of opportunity provision. Opportunity-enhancing practices within HCHRM, such as autonomy provision, team development, and WLB support, are therefore expected to moderate the effects of LMX and CWX by shaping the organizational conditions under which these social resources operate. By comparatively examining LMX and CWX in long-term care, the study aims to clarify the role of workplace social resources and assess, through multilevel analysis, how their effects are moderated by opportunity-enhancing practices.

### 2.5. Research Questions and Analytical Framework

**Figure 1:**
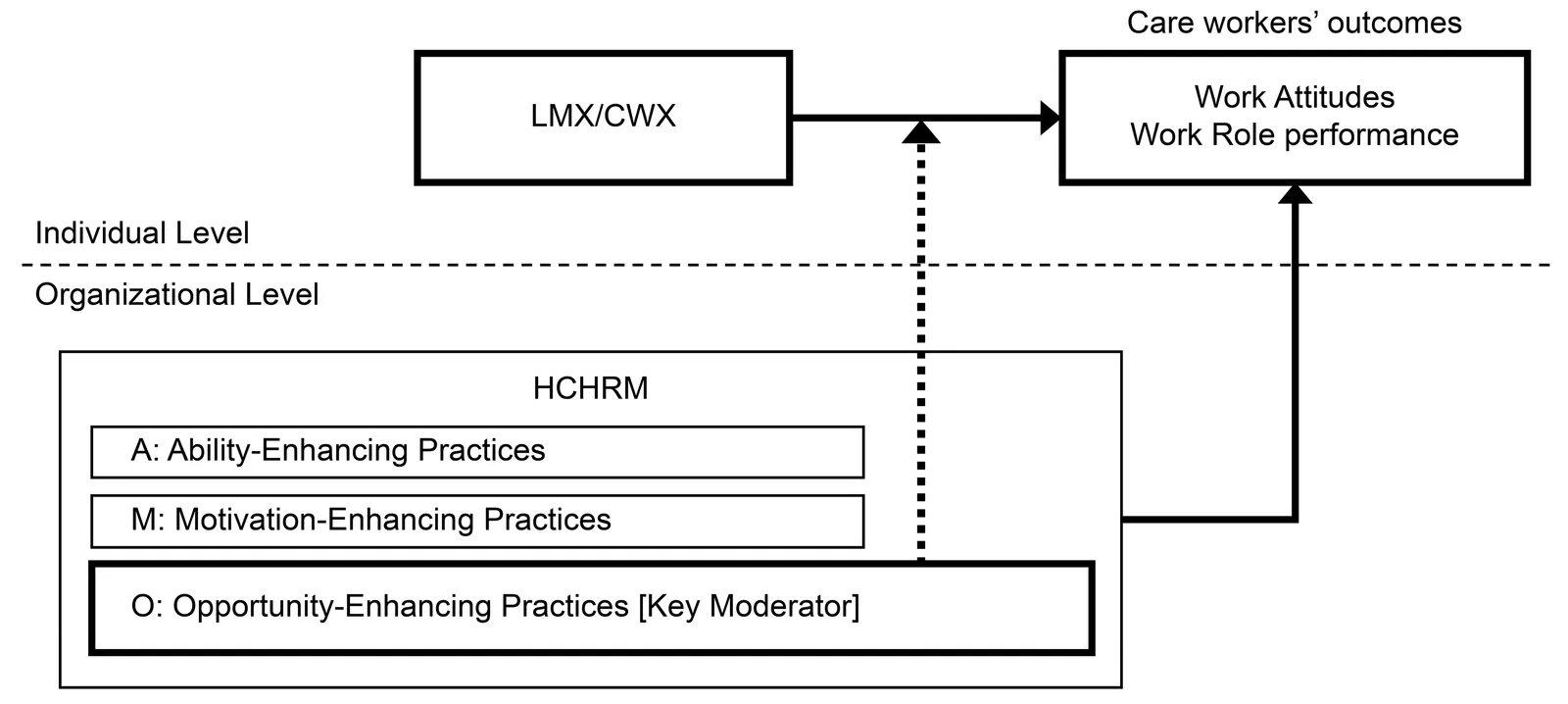
Analytical framework. Solid arrows indicate main effects; dashed arrows indicate cross-level interactions. Note. LMX = leader–member exchange; CWX = coworker exchange; HCHRM = high-commitment human resource management.

Building on the foregoing discussion, Figure 1 presents the analytical framework of this study.

This study examines how HCHRM incorporating WLB support (i.e., as an opportunity-enhancing component to reflect a sustainable HRM perspective) relates to work attitudes and work role performance among long-term care workers, examined through a sustainable-careers lens. AMO theory supports the general proposition that ability-, motivation-, and opportunity-enhancing practices contribute to employee outcomes; however, previous long-term care research provides little basis for predicting the direction or relative strength of individual AMO components, or for predicting whether opportunity-enhancing practices moderate LMX or CWX more strongly. Accordingly, rather than formulating directional confirmatory hypotheses that the available theory could not justify, I framed the direct effects as research questions and stated a general, non-confirmatory expectation for the cross-level moderation, as follows:

- RQ1: How does organizational-level HCHRM relate to care workers’ work attitudes and work role performance?
- RQ2: How do opportunity-enhancing practices within HCHRM moderate the relationships between individual-level social resources (LMX and CWX) and care workers’ work attitudes and work role performance?

Because WLB support is theoretically positioned as an opportunity-enhancing practice alongside the autonomy and discretion emphasized in previous research (Section 2.2), I expect that opportunity-enhancing practices, including WLB support, will moderate the relationships between social resources (LMX and CWX) and the outcomes, such that these relationships will be stronger where opportunity-enhancing practices are more strongly implemented. However, I was unable to formulate directional predictions about which social resource (LMX or CWX) will be moderated more strongly, or for which specific outcomes; these aspects were examined exploratorily.

## 3. Materials and Methods

### 3.1. Participants and Procedure

This study employs a multilevel (two-level) cross-sectional survey design, in which individual care workers (Level 1) were nested within establishments (Level 2). It targeted 10 corporations operating long-term care businesses in Japan (five for-profit and five non-profit). These corporations were recruited through the author’s research networks using purposive sampling, with the selection criteria ensuring variation in organizational size (100 or more employees), type, and geographic distribution. This approach was appropriate for three reasons. First, multilevel analysis requires a sufficient number of Level 2 units with multiple respondents per unit, making prior confirmation of organizational cooperation essential for data collection. Second, purposive sampling is widely used in HRM research requiring organizational access owing to the practical and ethical constraints associated with random participation (e.g., Renkema et al., 2021; Schopman et al., 2015). Third, securing variety in organizational type, size, and geographic distribution broadens the analytical scope. As random sampling was not employed, generalizability is limited.

Following Purcell and Hutchinson (2007), this study treats employees’ perceptions as indicators of HRM practices, collecting related data between September 2023 and October 2024 using a web-based, anonymous, self-administered questionnaire distributed to full-time care workers. Each corporation received a two-week response period followed by a one-week follow-up. Data collection proceeded sequentially, resulting in an overall survey period of approximately one year.

Each corporation operates multiple establishments, and although HRM policies may be set centrally, implementation occurs at the establishment level. Accordingly, establishments were treated as the unit of analysis, as HRM practices vary across them (Gerhart et al., 2000). Of 974 respondents from 188 establishments, those from establishments with fewer than three respondents were excluded (resulting in the exclusion of 58 establishments and 99 respondents), reflecting both the small-scale nature of long-term care settings and the minimum threshold required to estimate within-group variance (Ishiyama, 2020). The final sample comprised 875 workers from 130 establishments, with an average of 6.7 respondents per establishment (minimum: 3, maximum: 27). The dataset contained no missing values. As the survey was administered through organizations rather than a commercial panel, respondent motivation was expected to be relatively high.

Regarding sample size, a simulation study by Maas and Hox (2005) showed that with 100 Level 2 units, estimates of regression coefficients, variance components, and standard errors are accurate, and that the number of individuals per group has minimal impact on estimation precision. As the Level 2 sample (130 establishments) exceeded this threshold, substantial standard error bias was unlikely, despite the modest average group size. Furthermore, intraclass correlation coefficients (ICC(1)) for all establishment-level variables exceeded the recommended threshold (observed range 0.13–0.21), supporting the reliability of group means as indicators of group-level characteristics even with limited respondents per group (James, 1982).

Participant characteristics were as follows. By gender, 55.7% were female (*n* = 487), 41.8% male (*n* = 366), and 2.5% other (*n* = 22). By age group, 18.5% were in their 20s or younger (*n* = 162), 24.9% in their 30s (*n* = 218), 23.2% in their 40s (*n* = 203), 23.1% in their 50s (*n* = 202), and 10.3% aged 60 or older (*n* = 90). By position, 67.9% held non-leader roles (*n* = 594) and 32.1% held leader roles (*n* = 281). To assess the representativeness of the sample, I compared its composition with national statistics for care workers. The age distribution broadly corresponded to the national pattern (Ministry of Health, Labour and Welfare, 2025), and the proportion of leaders relative to non-leaders was consistent with the hierarchical structure of the sector. Men were partially overrepresented compared to the national proportion (41.8% vs. 34.7%). Overall, the sample broadly reflects the national long-term care workforce, although the modest overrepresentation of men should be borne in mind when interpreting the results.

This study was approved by the Research Ethics Review Committee of Ibaraki Christian University (Approval No. 2023-014; approval date: 16 June 2023) and conducted in accordance with the Declaration of Helsinki.

### 3.2. Measures

All measures were rated on a five-point Likert scale ranging from 1 (strongly disagree) to 5 (strongly agree). Table 1 (establishment-level variables) and Table 2 (individual-level variables) present descriptive statistics, reliability coefficients (Cronbach’s alpha), construct validity indices, and inter-factor correlations.

#### 3.2.1. HCHRM Practices

Drawing on the AMO framework (Appelbaum et al., 2000) and scale definitions by Lepak et al. (2006) and Edgar et al. (2021), items were adapted to Japanese long-term care settings. Specifically, the following subscales were developed: (A) training and development (four items: on-the-job training, internal training programs, support for external training, and qualification support); (M) organizational incentives (three items: redistribution of the Care Worker Treatment Improvement Allowance, salary levels relative to competitors, and performance-based compensation) and individual incentives (three items: performance-, evaluation-, and skill-based pay); and (O) autonomous team management (four items: flat organizational structure, on-site discretion, team self-management, and fair treatment regardless of employment status). Additionally, based on Berg et al. (2003) and Batt and Valcour (2003), WLB support (three items: WLB, ease of taking leave, and limited overtime) was included as an opportunity-enhancing (O) practice.

Exploratory factor analysis (maximum likelihood extraction, Promax rotation, loadings ≥ 0.40) confirmed the hypothesized five-factor structure. A confirmatory factor analysis (CFA) indicated acceptable fit (χ^2^(109) = 715.6, comparative fit index (CFI) = 0.908, Tucker–Lewis index (TLI) = 0.885, root mean square error of approximation (RMSEA) = 0.080, 90% CI [0.074, 0.085], standardized root mean squared residual (SRMR) = 0.052). To verify that the items reflected distinct constructs, I compared this five-factor model with a one-factor model, which fit substantially worse (Δχ^2^(10) = 1879.9, *p* < 0.001; ΔCFI = 0.284), supporting the five-factor structure (Table A3). Composite reliability (CR = 0.763–0.826) and average variance extracted (AVE = 0.531–0.572) exceeded the recommended thresholds (Fornell & Larcker, 1981). Discriminant validity was confirmed using the Fornell–Larcker criterion (Table 1). Table A1 reports scale items and standardized factor loadings.

#### 3.2.2. LMX and CWX

Given the high emotional demands of long-term care work, psychological support from supervisors and coworkers represents an important social resource. Accordingly, LMX was measured using three items from the 12-item LMX-MDM scale (Liden & Maslyn, 1998), capturing emotional bonds (affect), a trusting relationship that tolerates mistakes (loyalty), and willingness to contribute (professional respect). CWX was measured using three parallel items based on Sherony and Green (2002), with the referent adapted from a specific supervisor to “members of the establishment.” Although CWX originally refers to dyadic relationships, long-term care work is fundamentally team-based, requiring coordination across multiple coworkers and roles. Therefore, the referent was broadened to capture overall coworker relationship quality within the establishment, reflecting the “quality of affective relationships” as a conceptual counterpart to LMX. Accordingly, in this study CWX is best understood as perceived coworker relationship quality at the establishment level, rather than as a dyadic coworker exchange in its original sense.

Exploratory factor analysis supported a two-factor structure for LMX and CWX, while the CFA yielded a good fit (χ^2^(8) = 139.0, CFI = 0.966, TLI = 0.936, SRMR = 0.034). Although RMSEA = 0.137, 90% CI [0.117, 0.157], was elevated, it possibly reflects the inflation of RMSEA in models with small degrees of freedom (df = 8; Kenny et al., 2015), whereas the incremental indices (CFI, TLI) and SRMR indicated good fit. A one-factor model fit substantially worse (Δχ^2^(1) = 896.0, *p* < 0.001; ΔCFI = 0.233), supporting the two-factor structure (Table A3). All standardized loadings were ≥0.78. The CR (LMX = 0.90, CWX = 0.90) and AVE (LMX = 0.76, CWX = 0.75) for both constructs exceeded the recommended thresholds. The square roots of AVE (0.87 and 0.86) exceeded the inter-factor correlation (0.66), confirming discriminant validity via the Fornell–Larcker criterion (Table 2).

#### 3.2.3. Work Attitudes

Affective commitment was measured using four items assessing attachment to and identification with the organization, adapted from Allen and Meyer (1996). Turnover intention was measured using two items assessing intent to leave, adapted from Mobley et al. (1979) and Kishida and Tanigaki (2013).

Exploratory factor analysis confirmed a two-factor structure, while CFA yielded a good fit (χ^2^(8) = 116.3, CFI = 0.970, TLI = 0.944, SRMR = 0.044). Although RMSEA = 0.124, 90% CI [0.105, 0.145], was elevated, this possibly reflects the inflation of RMSEA in models with small degrees of freedom, as previously noted. A one-factor model fit worse (Δχ^2^(1) = 221.4, *p* < 0.001; ΔCFI = 0.060), supporting the two-factor structure (Table A3). The CR (affective commitment = 0.93, turnover intention = 0.72) and AVE (affective commitment = 0.78, turnover intention = 0.56) exceeded the recommended thresholds. The inter-factor correlation was −0.53, consistent with the theoretically expected negative association, and the square roots of AVE (0.88 and 0.75) exceeded the absolute value of the inter-factor correlation, confirming discriminant validity (Table 2).

#### 3.2.4. Work Role Performance

Three items each were used to measure the contributing behaviors in Griffin et al.’s (2007) work role performance model: proficiency, adaptive, and proactive behavior. Although Griffin et al. (2007) distinguished three levels of contribution targets (individual, team, and organization), team-level items were used given the team-based nature of long-term care work. Exploratory factor analysis indicated that adaptive and proactive behavior loaded onto a single factor. Griffin et al. (2007) also reported moderate-to-high correlations between these behaviors, and responding to situational changes and anticipating future needs tend to form a continuous process in interpersonal service work, including long-term care. Therefore, this study treats the integrated construct as “adaptive-proactive behavior,” yielding two subscales alongside proficiency behavior.

The CFA indicated acceptable fit (χ^2^(26) = 409.1, CFI = 0.916, TLI = 0.884, SRMR = 0.067). Although RMSEA = 0.130, 90% CI [0.119, 0.141], was elevated, this possibly reflects the inflation of RMSEA in models with small degrees of freedom, as previously noted. A one-factor model fit substantially worse (Δχ^2^(1) = 420.3, *p* < 0.001; ΔCFI = 0.092), supporting the two-factor structure (Table A3). The CR (proficiency behavior = 0.77, adaptive-proactive behavior = 0.91) and AVE (proficiency behavior = 0.54, adaptive-proactive behavior = 0.63) exceeded the recommended thresholds. The inter-factor correlation was 0.58, and the square roots of AVE (0.73 and 0.79) exceeded this value, confirming discriminant validity (Table 2). Table A2 presents all individual-level scale items and standardized factor loadings.

#### 3.2.5. Control Variables

Gender, age group, and position (leader vs. non-leader) were included as control variables.

### 3.3. Analytical Approach

Data were analyzed using SPSS Statistics Version 29. Hierarchical linear modeling (HLM) estimation used the mixed models procedure in SPSS with maximum likelihood estimation.

#### 3.3.1. Justification for Data Aggregation

To assess whether each HCHRM practice could be aggregated to the establishment level, ICC(1) values were calculated. The resulting values were as follows: training and development = 0.20, organizational incentives = 0.21, individual incentives = 0.13, autonomous team management = 0.13, and WLB support = 0.19. All exceeded the recommended threshold of 0.12 (James, 1982). ICC(2) values ranged from 0.49 to 0.64, and the median *r*_wg(j)_ ranged from 0.77 to 0.89 across practices, indicating acceptable within-group agreement (James et al., 1984; LeBreton & Senter, 2008). Although some ICC(2) values were modest, this is expected considering the relatively small average group size (*k*_avg_ = 6.7); the convergence of the three indices supports aggregation to the establishment level (Bliese, 2000). Table A4 presents full aggregation statistics.

#### 3.3.2. Justification for HLM

Null models were estimated for each dependent variable. The ICC(1) values were as follows: affective commitment = 0.12, turnover intention = 0.11, proficiency behavior = 0.06, and adaptive-proactive behavior = 0.04. As intercept variance components were statistically significant in all cases, HLM was statistically justified. The relatively lower ICC(1) values for behavioral outcomes likely reflect the tendency for attitudinal variables to capture shared workplace context and therefore exhibit greater between-group variance (e.g., Berthelsen et al., 2018; R. Takeuchi et al., 2009), whereas behavioral variables are more influenced by individual characteristics. The significance of variance components provides the primary justification for HLM, and the lower ICC values are consistent with behavioral outcomes.

#### 3.3.3. Centering and Common Method Bias

Level 1 variables (LMX and CWX) were group-mean centered, and Level 2 variables (HCHRM) were grand-mean centered (Enders & Tofighi, 2007). To assess common method bias, Harman’s single-factor test (Podsakoff & Organ, 1986) was conducted. The first factor accounted for 30.6% of the variance, below the 50% threshold. However, given the statistical limitations of this test (Podsakoff et al., 2003), procedural remedies were also employed: respondent anonymity was ensured to encourage candid responses (Podsakoff et al., 2003, 2012). Nonetheless, reliance on self-report data collected at a single time point remains a limitation.

#### 3.3.4. Analytical Procedure

To address RQ1 (main effects), intercepts-as-outcomes models (Hofmann, 1997) were estimated for each dependent variable. Control variables were entered in Model 1, Level 1 variables (LMX and CWX) in Model 2, and Level 2 variables (HCHRM) in Model 3. To address RQ2 (cross-level interactions), slopes-as-outcomes models (Hofmann, 1997) were estimated, with random slopes specified for LMX and CWX in Model 4. Model fit (−2 log-likelihood [−2LL], Akaike information criterion [AIC], and explained variance [marginal R^2^, conditional R^2^]; Nakagawa & Schielzeth, 2013) was compared with preceding models. Guided by the theoretical expectation stated in Section 2.5, opportunity-enhancing practices (WLB support and autonomous team management) were of primary interest; nevertheless, I tested cross-level interactions between all five HCHRM practices and each of the two social resources (LMX and CWX). For each outcome, random slopes were first tested (Model 4), and cross-level interactions were examined only where the random-slope variance was significant. To conserve space, only the significant interactions are reported in Table 3 and Table 4; all other interaction terms were non-significant. Considering the theory-driven and confirmatory-to-exploratory nature of these analyses, I did not apply a multiple-comparison correction, and interpreted the interaction findings as suggestive rather than definitive. Where interaction terms were significant, simple slope analyses were conducted following Aiken and West (1991). Notably, HLM explicitly accounts for the non-independence of errors within groups by modeling nested data structures, making it appropriate for individual-level observations nested within establishments.

## 4. Results

Table 3 and Table 4 present results for work attitudes and work role performance (proficiency behavior and adaptive-proactive behavior), respectively. Unstandardized coefficients are reported throughout.

### 4.1. Main Effects of HCHRM Practices and Social Resources (LMX/CWX)

To address RQ1, intercepts-as-outcomes models were constructed and estimated.

#### 4.1.1. Main Effects on Affective Commitment

The transition from Model 1 (control variables) to Model 2 (adding LMX and CWX) substantially improved marginal R^2^, from 0.02 to 0.26 (−2LL: 2316.93 → 2019.05). Both LMX (γ = 0.32, SE = 0.04, *p* < 0.001) and CWX (γ = 0.39, SE = 0.04, *p* < 0.001) showed significant positive effects. In Model 3 (adding HCHRM), WLB support (γ = 0.26, SE = 0.07, *p* < 0.001), autonomous team management (γ = 0.27, SE = 0.09, *p* < 0.01), and individual incentives (γ = 0.26, SE = 0.09, *p* < 0.01) each showed significant positive effects. Model 3 explained marginal R^2^ = 0.40 and conditional R^2^ = 0.43 of the variance. Regarding individual characteristics, affective commitment was significantly higher among workers aged 40 and above than among those in their 20s or younger.

#### 4.1.2. Main Effects on Turnover Intention

In Model 2, both LMX (γ = −0.15, SE = 0.05, *p* < 0.001) and CWX (γ = −0.16, SE = 0.05, *p* < 0.001) showed significant negative effects. In Model 3, organizational incentives (γ = −0.19, SE = 0.09, *p* < 0.05) was the only Level 2 variable to show a significant negative effect. Model 3 explained marginal R^2^ = 0.12 and conditional R^2^ = 0.18 of the variance. Regarding individual characteristics, turnover intention was significantly lower across all age groups than among workers in their 20s or younger.

#### 4.1.3. Main Effects on Proficiency Behavior

In Model 2, both LMX (γ = 0.11, SE = 0.03, *p* < 0.001) and CWX (γ = 0.29, SE = 0.03, *p* < 0.001) showed significant positive effects, with CWX being stronger. In Model 3, autonomous team management (γ = 0.25, SE = 0.06, *p* < 0.001) and training and development (γ = 0.15, SE = 0.06, *p* < 0.05) showed significant positive effects, while organizational incentives (γ = −0.10, SE = 0.05, *p* < 0.05) showed a significant negative effect. Model 3 explained marginal R^2^ = 0.25 and conditional R^2^ = 0.28 of the variance. Gender showed a significant effect in Model 2 (γ = −0.07, *p* < 0.05) but became non-significant upon entry of Level 2 variables in Model 3.

#### 4.1.4. Main Effects on Adaptive-Proactive Behavior

In Model 2, only CWX (γ = 0.25, SE = 0.04, *p* < 0.001) showed a significant positive effect, whereas the effect of LMX was not significant (γ = 0.03, SE = 0.04, n.s.). In Model 3, no establishment-level variables were significant. Model 3 explained marginal R^2^ = 0.14 and conditional R^2^ = 0.16 of the variance. Adaptive-proactive behavior was higher among workers in their 30s and above, among leaders, and among male participants.

### 4.2. Cross-Level Interactions of Opportunity-Enhancing Practices

To address RQ2, slopes-as-outcomes models were specified based on Model 3, with random slopes specified for LMX and CWX (Model 4). Cross-level interaction analyses were conducted for outcomes showing an improved model fit and explained variance.

#### 4.2.1. Interactions on Affective Commitment

Model 4, which included random slopes for LMX and CWX, showed a poorer fit (AIC: 1943.11 → 1950.82); therefore, cross-level interactions were not examined.

#### 4.2.2. Interactions on Turnover Intention

**Figure 2:**
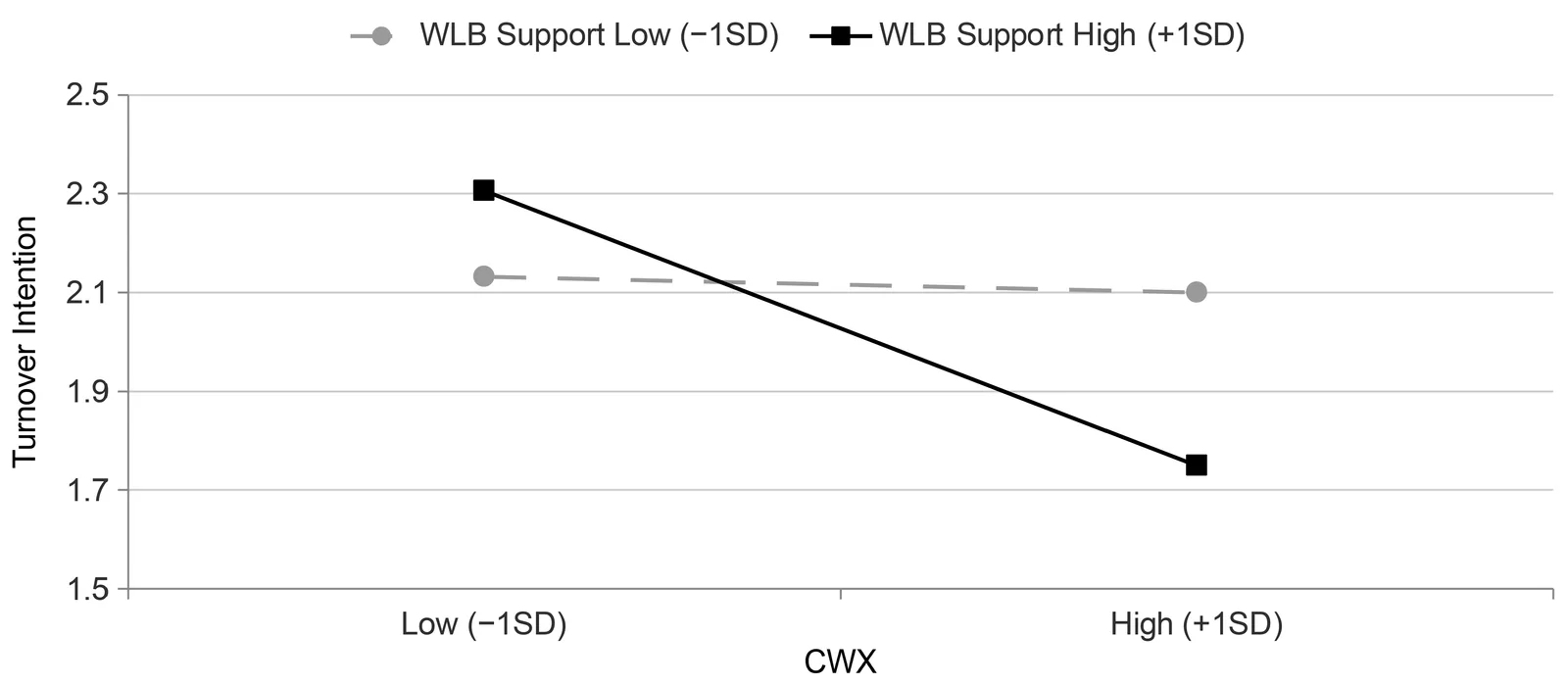
Interaction effect of CWX × WLB support on turnover intention. Simple slope analysis depicts the relationships at ±1 SD. Note. CWX = coworker exchange; WLB = work–life balance.

Specifying random slopes in Model 4 improved model fit (AIC: 2287.22 → 2265.06), and variance components were significant; therefore, cross-level interactions were examined. In Model 5, the LMX × WLB support interaction (γ = −0.25, SE = 0.09, *p* < 0.01) was significant; in Model 6, the CWX × WLB support interaction (γ = −0.37, SE = 0.10, *p* < 0.001) was significant. Both interactions were negative. Comparing Models 5 (AIC = 2259.90, conditional R^2^ = 0.31) and 6 (AIC = 2254.44, conditional R^2^ = 0.30), Model 6 was selected based on a lower AIC, and simple slope analysis was conducted (Figure 2). When WLB support was high (+1 SD), CWX was negatively associated with turnover intention (γ = −0.40, *p* < 0.001); when low (−1 SD), the association was not significant (γ = −0.02, *p* = 0.719). Simple slope analysis for Model 5 revealed a similar pattern: when WLB support was high (+1 SD), LMX was negatively associated with turnover intention (γ = −0.27, *p* < 0.001); when low (−1 SD), the association was not significant (γ = −0.08, *p* = 0.217).

#### 4.2.3. Interactions on Proficiency Behavior

Specifying random slopes in Model 4 improved model fit and yielded significant variance components (AIC: 1324.27 → 1324.00); cross-level interactions were therefore examined. In Model 5, the CWX × WLB support interaction showed a significant positive effect (γ = 0.13, SE = 0.05, *p* < 0.05; AIC = 1320.39, conditional R^2^ = 0.35). Simple slope analysis indicated that CWX was positively associated with proficiency behavior at both high (γ = 0.37, *p* < 0.001) and low (γ = 0.24, *p* < 0.001) levels of WLB support, with a stronger effect at higher levels.

#### 4.2.4. Interactions on Adaptive-Proactive Behavior

**Figure 3:**
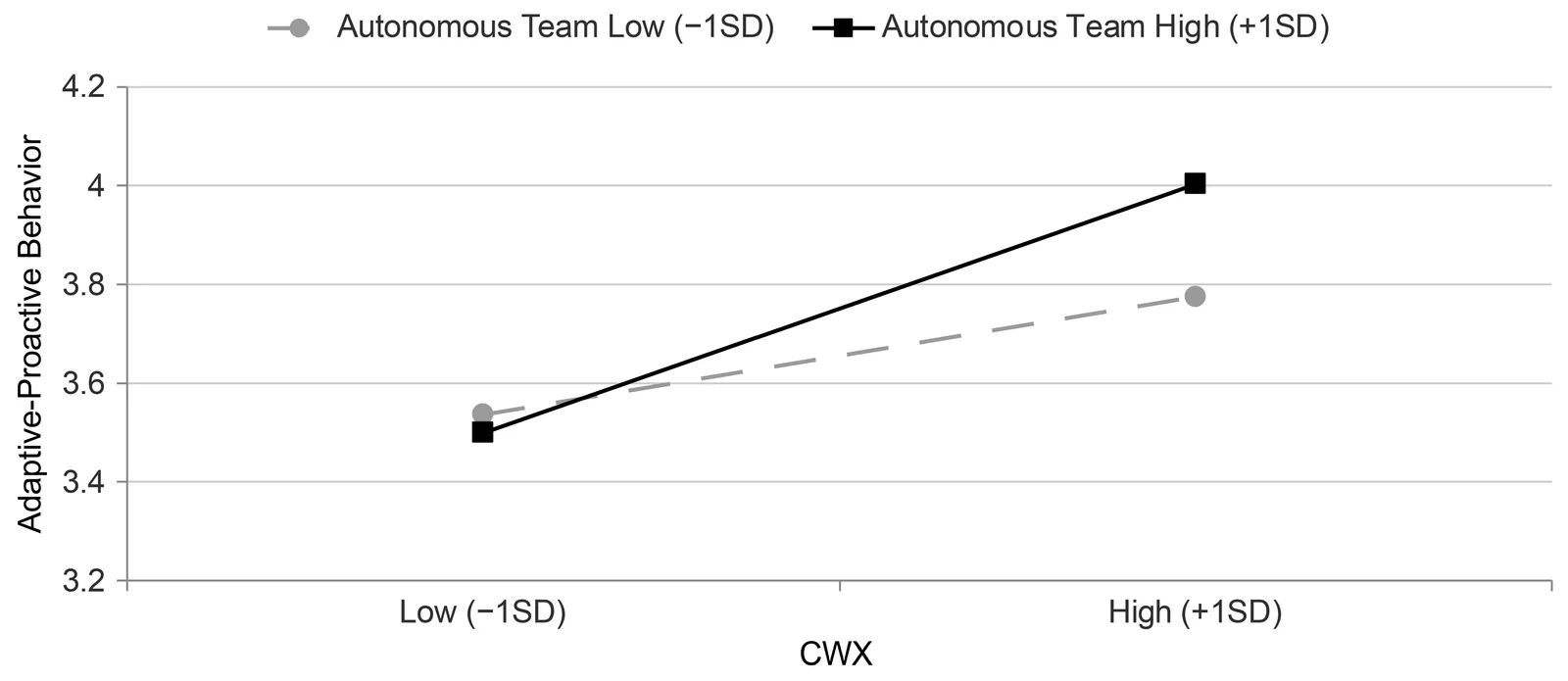
Interaction effect of CWX × autonomous team management on adaptive-proactive behavior. Simple slope analysis depicts the relationships at ±1 SD. Note. CWX = coworker exchange.

Specifying random slopes in Model 4 improved model fit and yielded significant variance components (AIC: 1932.35 → 1918.20); therefore, cross-level interactions were examined. In Model 5, the CWX × autonomous team management interaction showed a significant positive effect (γ = 0.24, SE = 0.11, *p* < 0.05; AIC = 1915.76, conditional R^2^ = 0.25). Simple slope analysis (Figure 3) indicated that CWX was significantly and positively associated with adaptive-proactive behavior at both high (γ = 0.36, *p* < 0.001) and low (γ = 0.17, *p* < 0.001) levels of autonomous team management, with a stronger effect at higher levels.

## 5. Discussion

This study examines, using multilevel analysis, how organizational HRM practices relate to work attitudes and work role performance among long-term care workers, viewed through a sustainable-careers lens, with HCHRM grounded in AMO theory as the framework.

### 5.1. RQ1: Main Effects of HCHRM

The findings for RQ1 indicate that opportunity-enhancing practices and individual-level social resources are associated with care workers’ outcomes, though the specific associations varied across practices and outcomes, as detailed below. First, opportunity-enhancing practices (O: autonomous team management and WLB support) and individual incentives (M) were positively associated with affective commitment, consistent with the findings of a meta-analysis by Jiang et al. (2012). The association of WLB support (O) with affective commitment suggests that opportunities for resource recovery and perceived control support sustained organizational attachment (Batt & Valcour, 2003; Hobfoll et al., 2018). Given that care workers often face irregular schedules and high task uncertainty requiring judgment without clear solutions, they may find it difficult to manage work–life boundaries. Organizational WLB support may therefore play a particularly important role in sustaining their affective commitment. However, notably, WLB support was associated with affective commitment but not directly with turnover intention or work role performance; as discussed in Section 5.2, its association with lower turnover emerged in conjunction with social resources rather than as a direct effect. The positive associations of autonomous team management and training and development with proficiency behavior align with Sugano’s (2020) and Jiang et al.’s (2012) findings.

Second, organizational-level associations were more pronounced for work attitudes, whereas individual-level associations were more prominent for work role performance. The absence of direct organizational associations with adaptive-proactive behavior suggests that these higher-order behaviors depend more on individual factors such as experience and role. In long-term care, workers must often respond flexibly within multidisciplinary teams to varying care needs and family expectations. This suggests that their judgment abilities may develop primarily through accumulated experience and ongoing coworker interactions. Relatedly, organizational-level practices were associated with affective commitment—an attitude possibly shared within establishments—but not directly with the more distal outcome of turnover intention. This gradient may suggest that organizational-level influences reach distal outcomes such as turnover intention indirectly, through more proximal and collectively shared attitudes such as affective commitment. I did not test such mediation directly, and this interpretation awaits future research using designs suited to estimating indirect effects.

Third, organizational incentives showed limited associations with the outcomes, including a weak negative association with turnover intention, no significant association with affective commitment, and a weak negative association with proficiency behavior. These associations were small in magnitude, and the measure captured workers’ perceptions of incentive practices rather than objective compensation levels or policy-level financial incentives. Therefore, I have refrained from an unnecessarily stringent and strong interpretation; although the pattern is consistent with a possible crowding-out of intrinsic motivation by extrinsic rewards (Frey & Jegen, 2001), this mechanism was not directly tested and remains a question for future research. Nonetheless, the limited associations of organizational incentives, together with the more consistent associations of opportunity-enhancing practices, are consistent with the broader argument that approaches relying primarily on government-led wage measures may be insufficient on their own.

### 5.2. RQ2: Cross-Level Interactions Between Opportunity-Enhancing Practices and Social Resources

The analyses addressing RQ2 indicated that the associations involving horizontal CWX were stronger under higher levels of organizational opportunity provision for work attitudes and work role performance among long-term care workers. First, CWX showed significant associations across outcomes, whereas LMX associations were more limited. This finding diverges from those in the study by Sherony and Green (2002), who reported stronger effects for LMX. Although this result may partly reflect the collectivistic characteristics of Japanese society, it more directly implies the distinctive nature of long-term care work. Specifically, the 24-h shift system creates temporal and physical separation between supervisors and subordinates, limiting direct supervisory support, while the extremely high task interdependence among workers requires close coworker coordination. This can explain why CWX, reflecting relationships with proximate and psychologically connected coworkers, emerged as a stronger predictor of attitudes and behavior, potentially reflecting the structural characteristics of long-term care work.

Second, the associations involving social resources were stronger under higher levels of opportunity-enhancing practices, and the pattern differed by outcome. These findings support AMO theory, which posits that opportunity-enhancing practices (O) function as conditions that moderate the contribution of individual ability and motivation (Blumberg & Pringle, 1982; Bos-Nehles et al., 2023). Regarding turnover intention, WLB support moderated the associations of both LMX and CWX, indicating that opportunities for resource recovery may strengthen the retention-related benefits of supportive workplace relationships in general, whether vertical or horizontal. Regarding work role performance, by contrast, the moderating associations were confined to CWX: higher WLB support was associated with a partially stronger link between CWX and proficiency behavior, and higher autonomous team management with a partially stronger link between CWX and adaptive-proactive behavior. This contrast implies that, whereas resource recovery may broadly reinforce the relational basis of retention, the activation of higher work role performance appears to depend more specifically on horizontal coworker relationships in team-based care. These patterns are consistent with the idea that, in team-based care, WLB support may generate psychological capacity that workers can direct toward coworker relationships (Hobfoll et al., 2018). These patterns, interpreted through a sustainable-careers lens, can be understood as relating to the core elements emphasized in that literature: long-term employment continuity and adaptive engagement with change.

I have further noted that WLB support was associated with affective commitment—and, through cross-level interactions, with retention- and performance-related outcomes—even though men were partially overrepresented in the present sample relative to the predominantly female care workforce. Because WLB needs are sometimes assumed to be more salient among women, who more often carry domestic and caregiving responsibilities, the emergence of these associations in a comparatively male-skewed sample may indicate that the relevance of WLB support is not confined to a particular gender group. However, this interpretation remains tentative as the study did not test gender differences in these associations directly.

### 5.3. Theoretical and Practical Implications

This study makes three main theoretical contributions. First, it applies multilevel analysis to the long-term care sector, where empirical HRM research remains limited, and clarifies how organizational-level HCHRM relates to care workers’ outcomes. Second, the study positions WLB support as an opportunity component within AMO theory and examines its moderating role in relation to CWX. This contributes to sustainable HRM research (De Vos & Van der Heijden, 2017) by implying that resource recovery and autonomy may strengthen the role of interpersonal resources. Third, it highlights the central role of CWX in long-term care settings.

Three practical implications follow from these findings. First, organizations should introduce and promote WLB support practices, such as reducing excessive working hours, encouraging paid leave use, and supporting the balance of work and family responsibilities. These practices may be particularly effective in combination with supportive coworker relationships, as indicated by the cross-level patterns observed here, making it essential to cultivate an organizational climate where they are actively utilized. Second, promoting autonomous team management, such as through delegation of authority and support for team-level decision-making, is recommended. This approach was associated with proactive work behavior—particularly in combination with strong coworker relationships—but requires a shift away from top-down organizational cultures. Third, alongside these structural supports, organizations should foster a workplace climate that nurtures positive coworker relationships. Given the interpersonal demands of long-term care work, where stress and conflict are prone to arise, organizations should not rely solely on individual effort but instead create supportive environments through structured practices such as facilitating open dialogue, conducting regular check-ins, and improving information sharing and team meetings.

### 5.4. Limitations and Future Directions

Several limitations should be noted. First, because the analyses were based on cross-sectional data, causal relationships cannot be established, and reverse causality cannot be ruled out. For example, workers with higher affective commitment may evaluate HRM practices more favorably, so the observed associations may partly operate in the opposite direction. Longitudinal research is needed to enable causal inferences. Relatedly, this study analyzed each outcome separately and did not test mediation; as discussed in Section 5.1, whether organizational-level practices reach distal outcomes indirectly through more proximal shared attitudes remains an important direction for future research using designs suited to estimating indirect effects. Second, data were collected exclusively from long-term care establishments in Japan, limiting generalizability. Additionally, because the sample was obtained through purposive rather than probability sampling, classical sampling error cannot be quantified, and the sample may not be representative of the broader long-term care workforce; therefore, these features represent threats to external validity, and the findings should be generalized carefully.

Third, the use of self-reported measures at a single time point means that common method bias cannot be entirely ruled out. Furthermore, as perceptions of HRM practices vary across individuals, perceptual bias may have been present. Fourth, the statistical results should be interpreted carefully. The proportion of variance explained varied across outcomes (marginal R^2^ ≈ 0.12–0.40), and the incremental variance explained by the cross-level interactions was modest; therefore, the interaction findings should be interpreted as indicating the presence of moderation rather than as accounting for a large share of variance. In addition, although ICC(1), ICC(2), and *r*_wg(j)_ jointly supported aggregation, some ICC(2) values were modest and the average group size was small (*k*_avg_ = 6.7); the establishment-level estimates should therefore be treated with corresponding caution. More generally, regarding statistical conclusion validity, the reliance on single-source, single-time-point data and the testing of multiple interaction terms without formal correction may affect the robustness of certain estimates. The cross-level interaction findings are therefore best regarded as suggestive rather than definitive, and replication with confirmatory designs is needed.

Fifth, some limitations relate to measurement. Regarding CWX, the original conceptualization by Sherony and Green (2002) refers to dyadic relationships with specific coworkers, whereas this study broadens the referent to coworkers within the establishment to reflect team-based care. Although this modification improved contextual fit, it may have affected construct validity given its departure from the original conceptualization. Accordingly, findings involving CWX should be understood as pertaining to perceived coworker relationship quality at the establishment level, rather than to dyadic coworker exchange as originally conceptualized. Despite acceptable CFA results (CR = 0.90, AVE = 0.75), further validation in other contexts is warranted. Similarly, the HCHRM scale was adapted to Japanese long-term care practice, and its generalizability to other settings requires caution. Lastly, exploratory factor analysis indicated that adaptive and proactive behavior converged onto a single factor, which warrants further investigation.

## 6. Conclusions

In Japan, a super-aged society, supporting sustainable careers among long-term care workers is an urgent priority. Using multilevel analysis, this study highlights the relevance of organizational opportunity provision for related goals. To support sustainable careers, organizations should provide opportunities for resource recovery and perceived control through WLB support, discretionary opportunities through autonomous team management, and should cultivate environments where these practices operate jointly with workplace relationships. The findings indicate that opportunity-enhancing practices may be associated with care workers’ affective commitment and, combined with the quality of workplace relationships, may further relate to lower turnover intention and more proactive behavior at work.

## Figures and Tables

**Table 1:** Descriptive statistics, reliability, validity, and inter-factor correlations for establishment-level variables.

Variable	M	SD	α	CR	AVE	1	2	3	4	5
1. Training and development	3.19	0.27	0.82	0.83	0.54	(0.74)				
2. Autonomous team management	3.50	0.29	0.82	0.82	0.54	0.516 ***	(0.73)			
3. Organizational incentives	3.04	0.27	0.79	0.79	0.56	0.562 ***	0.257 **	(0.75)		
4. Individual incentives	2.98	0.28	0.81	0.80	0.57	0.600 ***	0.287 ***	0.563 ***	(0.76)	
5. Work–life balance (WLB) support	3.30	0.29	0.73	0.76	0.53	0.449 ***	0.531 ***	0.245 **	0.233 **	(0.73)

*N* = 130. Values in parentheses on the diagonal represent the square root of average variance extracted (√AVE). Discriminant validity was confirmed using the Fornell–Larcker criterion (√AVE > inter-factor correlations). M = mean; SD = standard deviation; α = Cronbach’s α; AVE = average variance extracted; CR = composite reliability. *** *p* < 0.001, ** *p* < 0.01.

**Table 2:** Descriptive statistics, reliability, validity, and inter-factor correlations for individual-level variables.

Variable	M	SD	α	CR	AVE	1	2	3	4	5	6
1. Leader–member exchange (LMX)	3.70	0.49	0.91	0.90	0.76	(0.87)					
2. Coworker exchange (CWX)	3.74	0.43	0.90	0.90	0.75	0.600 ***	(0.86)				
3. Affective commitment	3.32	0.46	0.93	0.93	0.78	0.512 ***	0.525 ***	(0.88)			
4. Turnover intention	2.54	0.95	0.72	0.72	0.56	−0.263 ***	−0.269 ***	−0.466 ***	(0.75)		
5. Proficiency behavior	3.87	0.59	0.77	0.77	0.54	0.366 ***	0.499 ***	0.374 ***	−0.181 ***	(0.73)	
6. Adaptive-proactive behavior	3.26	0.77	0.91	0.91	0.63	0.206 ***	0.292 ***	0.331 ***	−0.131 ***	0.423 ***	(0.79)

*N* = 875. Values in parentheses on the diagonal represent the square root of average variance extracted (√AVE). Discriminant validity was confirmed using the Fornell–Larcker criterion (√AVE > inter-factor correlations). M = mean; SD = standard deviation; α = Cronbach’s α; AVE = average variance extracted; CR = composite reliability. *** *p* < 0.001.

**Table 3:** Multilevel analysis results for work attitudes. (**a**) Affective commitment. (**b**) Turnover intention.

(**a**)																									
**Variable**			**Affective Commitment**																						
			**Null**						**M1**					**M2**						**M3**					
			**γ**		**SE**		***p***		**γ**		**SE**	***p***		**γ**		**SE**		***p***		**γ**		**SE**		***p***	
Intercept		γ00	3.34		(0.04)		***		3.16		(0.08)	***		3.18		(0.07)		***		3.14		(0.07)		***	
**Control variables**																									
Gender (male vs. female)		γ10							0.02		(0.07)			−0.01		(0.05)				0.02		(0.05)			
Age (30s vs. 20s or younger)		γ20							0.06		(0.10)			0.06		(0.08)				0.07		(0.08)			
Age (40s vs. 20s or younger)		γ30							0.26		(0.10)	*		0.27		(0.08)		***		0.31		(0.08)		***	
Age (50s vs. 20s or younger)		γ40							0.13		(0.10)			0.17		(0.08)		*		0.24		(0.08)		**	
Age (60s or older vs. 20s or younger)		γ50							0.05		(0.12)			0.20		(0.10)				0.25		(0.10)		*	
Position (leader vs. non-leader)		γ60							0.17		(0.07)			0.08		(0.06)				0.04		(0.06)			
**Level 1 variables**																									
LMX		γ70												0.32		(0.04)		***		0.33		(0.04)		***	
CWX		γ80												0.39		(0.04)		***		0.39		(0.04)		***	
**Level 2 variables (high-commitment human resource management [HCHRM])**																									
Training and development		γ01																		0.17		(0.09)			
Autonomous team management		γ02																		0.27		(0.09)		**	
Organizational incentives		γ03																		0.11		(0.07)			
Individual incentives		γ04																		0.26		(0.09)		**	
WLB support		γ05																		0.26		(0.07)		***	
**Cross-level interaction terms**																									
Level 1 residual variance σ^2^			0.78				***		0.76			***		0.51				***		0.50				***	
Between-group variance in intercept τ_00_			0.10				***		0.10			***		0.14				***		0.03				*	
Deviance (−2LL)			2336.87						2316.93					2019.05						1911.11					
AIC			2342.87						2334.93					2041.05						1943.11					
Marginal R^2^			0.00						0.02					0.26						0.40					
Conditional R^2^			0.11						0.13					0.42						0.43					
(**b**)																									
**Variable**		**Turnover Intention**																							
		**Null**			**M1**			**M2**			**M3**				**M4**				**M5**			**M6**			
		**γ**	**SE**	***p***	**γ**	**SE**	***p***	**γ**	**SE**	***p***	**γ**	**SE**	***p***		**γ**	**SE**	***p***		**γ**	**SE**	***p***	**γ**	**SE**		***p***
Intercept	γ00	2.50	(0.04)	***	2.83	(0.08)	***	2.81	(0.08)	***	2.84	(0.08)	***		2.82	(0.08)	***		2.82	(0.08)	***	2.81	(0.08)		***
**Control variables**																									
Gender (male vs. female)	γ10				−0.03	(0.07)		0.02	(0.06)		0.03	(0.06)			0.02	(0.06)			0.02	(0.06)		0.02	(0.06)		
Age (30s vs. 20s or younger)	γ20				−0.36	(0.13)	**	−0.42	(0.12)	***	−0.46	(0.12)	***		−0.45	(0.12)	***		−0.45	(0.12)	***	−0.46	(0.12)		***
Age (40s vs. 20s or younger)	γ30				−0.36	(0.10)	***	−0.38	(0.10)	***	−0.42	(0.10)	***		−0.41	(0.09)	***		−0.42	(0.09)	***	−0.40	(0.09)		***
Age (50s vs. 20s or younger)	γ40				−0.40	(0.10)	***	−0.40	(0.10)	***	−0.42	(0.10)	***		−0.37	(0.09)	***		−0.37	(0.09)	***	−0.37	(0.09)		***
Age (60s or older vs. 20s or younger)	γ50				−0.22	(0.10)	*	−0.22	(0.09)	*	−0.23	(0.09)	*		−0.20	(0.09)	*		−0.20	(0.09)	*	−0.20	(0.09)		*
Position (leader vs. non-leader)	γ60				−0.09	(0.07)		−0.05	(0.07)		−0.03	(0.07)			−0.03	(0.07)			−0.04	(0.07)		−0.03	(0.07)		
**Level 1 variables**																									
LMX	γ70							−0.15	(0.05)	***	−0.16	(0.05)	***		−0.15	(0.05)	**		−0.17	(0.05)	**	−0.14	(0.05)		**
CWX	γ80							−0.16	(0.05)	***	−0.16	(0.05)	***		−0.18	(0.06)	**		−0.17	(0.06)	**	−0.21	(0.06)		***
**Level 2 variables (HCHRM)**																									
Training and development	γ01										0.03	(0.11)			0.00	(0.11)			0.00	(0.11)		0.01	(0.11)		
Autonomous team management	γ02										−0.17	(0.12)			−0.21	(0.11)			−0.21	(0.11)		−0.20	(0.11)		
Organizational incentives	γ03										−0.19	(0.09)	*		−0.22	(0.09)	*		−0.22	(0.09)	*	−0.22	(0.09)		*
Individual incentives	γ04										−0.17	(0.11)			−0.12	(0.11)			−0.12	(0.11)		−0.13	(0.11)		
WLB support	γ05										−0.11	(0.09)			−0.02	(0.08)			−0.06	(0.08)		−0.09	(0.08)		
**Cross-level interaction terms**																									
LMX × WLB support	γ75																		−0.25	(0.09)	**				
CWX × WLB support	γ85																					−0.37	(0.10)		***
Level 1 residual variance σ^2^		0.80		***	0.78		***	0.73		***	0.73		***		0.63		***		0.62		***	0.63			***
Between-group variance in intercept τ_00_		0.10		***	0.09		***	0.09		*	0.06		**		0.08		***		0.08		***	0.08			***
Deviance (−2LL)		2355.92			2330.41			2282.26			2255.22				2223.06				2215.90			2210.44			
AIC		2361.92			2348.41			2304.26			2287.22				2265.06				2259.90			2254.44			
Marginal R^2^		0.00			0.03			0.08			0.12				0.12				0.13			0.14			
Conditional R^2^		0.11			0.12			0.18			0.18				0.30				0.31			0.30			

Employees, *N* = 875; Establishments, *N* = 130. *** *p* < 0.001, ** *p* < 0.01, * *p* < 0.05. VIF = 1.386–2.172. SE = standard error. Coefficients are unstandardized.

**Table 4:** Multilevel analysis results for work role performance. (**a**) Proficiency behavior. (**b**) Adaptive-proactive behavior.

**(a)**																			
**Variable**		**Proficiency Behavior**																	
		**Null**			**M1**			**M2**			**M3**			**M4**			**M5**		
		**γ**	**SE**	***p***	**γ**	**SE**	***p***	**γ**	**SE**	***p***	**γ**	**SE**	***p***	**γ**	**SE**	***p***	**γ**	**SE**	***p***
Intercept	γ00	3.88	(0.02)	***	3.92	(0.05)	***	3.92	(0.05)	***	3.91	(0.05)	***	3.91	(0.05)	***	3.91	(0.05)	***
**Control variables**																			
Gender (male vs. female)	γ10				−0.07	(0.04)		−0.07	(0.04)	*	−0.07	(0.04)		−0.07	(0.04)	*	−0.07	(0.04)	*
Age (30s vs. 20s or younger)	γ20				−0.02	(0.06)		−0.02	(0.06)		−0.03	(0.05)		−0.03	(0.05)		−0.03	(0.05)	
Age (40s vs. 20s or younger)	γ30				−0.04	(0.06)		−0.03	(0.06)		−0.04	(0.06)		−0.04	(0.06)		−0.04	(0.06)	
Age (50s vs. 20s or younger)	γ40				0.04	(0.06)		0.05	(0.06)		0.07	(0.06)		0.07	(0.06)		0.07	(0.06)	
Age (60s or older vs. 20s or younger)	γ50				−0.03	(0.08)		0.04	(0.07)		0.05	(0.07)		0.05	(0.07)		0.05	(0.07)	
Position (leader vs. non-leader)	γ60				−0.01	(0.04)		−0.05	(0.04)		−0.05	(0.04)		−0.03	(0.04)		−0.03	(0.04)	
**Level 1 variables**																			
LMX	γ70							0.11	(0.03)	***	0.11	(0.03)	***	0.12	(0.03)	***	0.11	(0.03)	***
CWX	γ80							0.29	(0.03)	***	0.29	(0.03)	***	0.29	(0.03)	***	0.30	(0.03)	***
**Level 2 variables (HCHRM)**																			
Training and development	γ01										0.15	(0.06)	*	0.16	(0.06)	**	0.16	(0.06)	**
Autonomous team management	γ02										0.25	(0.06)	***	0.24	(0.06)	***	0.24	(0.06)	***
Organizational incentives	γ03										−0.10	(0.05)	*	−0.10	(0.05)	*	−0.10	(0.05)	*
Individual incentives	γ04										−0.03	(0.06)		−0.03	(0.06)		−0.03	(0.06)	
WLB support	γ05										0.06	(0.05)		0.05	(0.05)		0.05	(0.05)	
**Cross-level interaction terms**																			
CWX × WLB support	γ85																0.13	(0.05)	*
Level 1 residual variance σ^2^		0.33		***	0.32		***	0.25		***	0.25		***	0.23		***	0.23		***
Between-group variance in intercept τ_00_		0.02		*	0.02		*	0.03		***	0.01			0.01		*	0.01		*
Deviance (−2LL)		1543.03			1536.85			1337.82			1292.27			1282.00			1276.39		
AIC		1549.03			1554.85			1359.82			1324.27			1324.00			1320.39		
Marginal R^2^		0.00			0.01			0.19			0.25			0.26			0.27		
Conditional R^2^		0.06			0.07			0.28			0.28			0.35			0.35		
**(b)**																			
**Variable**	**Adaptive-Proactive Behavior**																		
		**Null**			**M1**			**M2**			**M3**			**M4**			**M5**		
		**γ**	**SE**	***p***	**γ**	**SE**	***p***	**γ**	**SE**	***p***	**γ**	**SE**	***p***	**γ**	**SE**	***p***	**γ**	**SE**	***p***
Intercept	γ00	3.27	(0.03)	***	2.91	(0.07)	***	2.91	(0.07)	***	2.89	(0.06)	***	2.90	(0.06)	***	2.90	(0.06)	***
**Control variables**																			
Gender (male vs. female)	γ10				0.09	(0.05)		0.10	(0.05)		0.10	(0.05)		0.10	(0.05)	*	0.10	(0.05)	
Age (30s vs. 20s or younger)	γ20				0.29	(0.08)	***	0.28	(0.08)	***	0.29	(0.08)	***	0.28	(0.08)	***	0.28	(0.07)	***
Age (40s vs. 20s or younger)	γ30				0.29	(0.08)	***	0.29	(0.08)	***	0.30	(0.08)	***	0.29	(0.08)	***	0.28	(0.08)	***
Age (50s vs. 20s or younger)	γ40				0.33	(0.08)	***	0.34	(0.08)	***	0.37	(0.08)	***	0.35	(0.08)	***	0.34	(0.08)	***
Age (60s or older vs. 20s or younger)	γ50				0.36	(0.10)	***	0.40	(0.10)	***	0.42	(0.10)	***	0.40	(0.10)	***	0.40	(0.10)	***
Position (leader vs. non-leader)	γ60				0.20	(0.06)	***	0.18	(0.06)	***	0.17	(0.06)	**	0.20	(0.05)	***	0.20	(0.05)	***
**Level 1 variables**																			
LMX	γ70							0.03	(0.04)		0.03	(0.04)		0.04	(0.04)		0.04	(0.04)	
CWX	γ80							0.25	(0.04)	***	0.25	(0.04)	***	0.25	(0.05)	***	0.27	(0.05)	***
**Level 2 variables (HCHRM)**																			
Training and development	γ01										0.14	(0.08)		0.13	(0.08)		0.13	(0.08)	
Autonomous team management	γ02										0.11	(0.08)		0.11	(0.08)		0.12	(0.08)	
Organizational incentives	γ03										−0.06	(0.07)		−0.07	(0.07)		−0.07	(0.07)	
Individual incentives	γ04										0.07	(0.08)		0.08	(0.08)		0.08	(0.08)	
WLB support	γ05										0.09	(0.06)		0.09	(0.06)		0.09	(0.06)	
**Cross-level interaction terms**																			
CWX × autonomous team management	γ82																0.24	(0.11)	*
Level 1 residual variance σ^2^		0.57		***	0.54		***	0.50		***	0.50		***	0.45		***	0.45		***
Between-group variance in intercept τ_00_		0.02		*	0.03		*	0.03		*	0.01			0.02		*	0.02		*
Deviance (−2LL)		2028.42			1981.88			1922.01			1900.35			1876.20			1871.76		
AIC		2034.42			1999.88			1944.01			1932.35			1918.20			1915.76		
Marginal R^2^		0.00			0.05			0.11			0.14			0.14			0.15		
Conditional R^2^		0.04			0.09			0.16			0.16			0.26			0.25		

Employees, *N* = 875; Establishments, *N* = 130. VIF = 1.386–2.172. SE = standard error. Coefficients are unstandardized. *** *p* < 0.001, ** *p* < 0.01, * *p* < 0.05.

## Data Availability

The data presented in this study are available on request from the corresponding author. The data are not publicly available owing to privacy or ethical restrictions.

## Abbreviations

The following abbreviations are used in this manuscript:

AIC	Akaike information criterion
AMO	ability–motivation–opportunity
AVE	average variance extracted
CFA	confirmatory factor analysis
CR	composite reliability
CWX	coworker exchange
HCHRM	high-commitment human resource management
HLM	hierarchical linear modeling
ICC	intraclass correlation coefficient
LMX	leader–member exchange
WLB	work–life balance

## Appendix A

**Table A1:** HCHRM scale items and standardized factor loadings.

Construct/Item	λ
**Training and development (α = 0.82, CR = 0.83, AVE = 0.54)**	
This workplace invests considerable time and money in developing human resources.	0.78
This workplace provides sufficient on-the-job training by supervisors/seniors and training programs.	0.80
This workplace actively supports obtaining official qualifications.	0.67
This workplace actively supports participation in external training and seminars.	0.69
**Organizational incentives (α = 0.79, CR = 0.79, AVE = 0.56)**	
This workplace offers higher salaries compared with competitors.	0.67
This workplace appropriately distributes the allowance for care worker treatment improvement.	0.74
Organizational- or group-level performance is reflected in salary increases or bonuses.	0.83
**Individual incentives (α = 0.81, CR = 0.80, AVE = 0.57)**	
Individual performance appraisal results affect wages (salaries and bonuses).	0.75
Even for the same job, wages differ based on individual performance.	0.70
Individual skill development and goal achievement are reflected in compensation.	0.81
**Autonomous team management (α = 0.82, CR = 0.82, AVE = 0.54)**	
Members can discuss and solve problems or make decisions on-site.	0.68
Team self-management and creativity are emphasized.	0.75
There is little hierarchy based on position or job type; it is relatively flat.	0.77
All employees, including non-regular staff, are treated fairly.	0.74
**WLB support (α = 0.73, CR = 0.76, AVE = 0.53)**	
There is little overtime or work on holidays.	0.51
It is easy to take paid leave or request specific days off.	0.70
The environment supports WLB, not limited to childcare or nursing care.	0.92

λ = standardized factor loading from CFA. α = Cronbach’s α; AVE = average variance extracted; CR = composite reliability. All loadings significant at *p* < 0.001.

**Table A2:** Individual-level variable items and standardized factor loadings.

Construct/Item	λ
**Leader–member exchange (LMX) (α = 0.91, CR = 0.90, AVE = 0.76)**	
I like my supervisor as a person.	0.87
My supervisor would defend me if I made an honest mistake.	0.83
I am willing to work hard for my current supervisor.	0.90
**Coworker exchange (CWX) (α = 0.90, CR = 0.90, AVE = 0.75)**	
I like the members of this workplace as people.	0.89
Members would defend me if I made an honest mistake.	0.79
I am willing to work hard with the current members.	0.91
**Affective commitment (α = 0.93, CR = 0.93, AVE = 0.78)**	
I feel emotionally attached to this organization.	0.91
I am proud to be a member of this organization.	0.94
I would tell friends that this is a good organization to work for.	0.87
I want to continue working for this organization as long as possible.	0.80
**Turnover intention (α = 0.72, CR = 0.72, AVE = 0.56)**	
I want to change jobs to another care organization.	0.76
I want to change jobs to another industry.	0.74
**Proficiency behavior (α = 0.77, CR = 0.77, AVE = 0.54)**	
I coordinate my work well with colleagues.	0.73
I communicate well with colleagues.	0.82
I help colleagues when asked or when needed.	0.64
**Adaptive-proactive behavior (α = 0.91, CR = 0.91, AVE = 0.63)**	
I effectively deal with changes that affect my workplace.	0.74
I learn new skills or take on new roles in preparation for changes.	0.69
I deal constructively with changes in how work is done.	0.71
I suggest ways to improve efficiency at the team level.	0.86
I develop or improve methods to enhance team performance.	0.88
I take initiative to improve work at the team level.	0.85

λ = standardized factor loading from CFA. All loadings significant at *p* < 0.001. LMX items were adapted from Liden and Maslyn (1998), while the CWX items from the LMX scale in the study by Sherony and Green (2002). Affective commitment items were adapted from the study by Allen and Meyer (1996), while behavioral items were adapted from the research conducted by Griffin et al. (2007).

**Table A3:** Confirmatory factor analyses: fit indices for the adopted measurement models and one-factor alternatives.

Model	χ^2^	df	CFI	TLI	RMSEA [90% CI]	SRMR	Δχ^2^ (Δdf)	ΔCFI
**HCHRM: 5-factor (adopted)**	**715.6**	**109**	**0.908**	**0.885**	**0.080 [0.074–0.085]**	**0.052**	**—**	**—**
HCHRM: 1-factor	2595.5	119	0.624	0.571	0.154 [0.149–0.159]	0.108	1879.9 (10) ***	0.284
**LMX/CWX: 2-factor (adopted)**	**139.0**	**8**	**0.966**	**0.936**	**0.137 [0.117–0.157]**	**0.034**	**—**	**—**
LMX/CWX: 1-factor	1035.0	9	0.733	0.555	0.361 [0.343–0.380]	0.116	896.0 (1) ***	0.233
**Work attitudes: 2-factor (adopted)**	**116.3**	**8**	**0.970**	**0.944**	**0.124 [0.105–0.145]**	**0.044**	**—**	**—**
Work attitudes: 1-factor	337.7	9	0.910	0.850	0.204 [0.186–0.223]	0.095	221.4 (1) ***	0.060
**Work role performance: 2-factor (adopted)**	**409.1**	**26**	**0.916**	**0.884**	**0.130 [0.119–0.141]**	**0.067**	**—**	**—**
Work role performance: 1-factor	829.4	27	0.824	0.765	0.184 [0.174–0.195]	0.101	420.3 (1) ***	0.092

Note. *N* = 875 (individual-level models) and *N* = 130 (HCHRM, establishment-level model). Bold rows indicate the adopted (hypothesized) models. Δχ^2^ and ΔCFI compare each one-factor alternative against the corresponding adopted model. SRMR and the incremental indices (CFI, TLI) are robust to the small degrees of freedom that inflate the RMSEA in the lower-df models. *** *p* < 0.001.

**Table A4:** Aggregation statistics for establishment-level HRM practices (*k*_avg_ = 6.7, range 3–27; *N* = 130 establishments).

HRM Practice	ICC(1)	ICC(2)	*r*_wg(j)_ Median	% Groups *r*_wg(j)_ ≥ 0.70
Training and development	0.20	0.63	0.86	84.6%
Autonomous team management	0.13	0.51	0.89	86.2%
Organizational incentives	0.21	0.64	0.80	73.1%
Individual incentives	0.13	0.49	0.83	78.5%
WLB support	0.19	0.61	0.77	61.5%

Note. ICC(1) and ICC(2) were computed from one-way ANOVA (Bliese, 2000). *r*_wg(j)_ was calculated assuming a uniform null distribution (σ^2^EU = 2.00 for 5-point scales; James et al., 1984). The 0.70 criterion follows LeBreton and Senter (2008).
